# Access to HIV care and treatment for migrants between Lesotho and South Africa: a mixed methods study

**DOI:** 10.1186/s12889-018-5594-3

**Published:** 2018-05-29

**Authors:** Iyiola Faturiyele, Dimitris Karletsos, Keletso Ntene-Sealiete, Alfred Musekiwa, Mantiti Khabo, Marethabile Mariti, Phetole Mahasha, Thembisile Xulu, Pedro T. Pisa, on behalf of EQUIP Innovation for Health Team

**Affiliations:** 1 EQUIP-Innovation for Health, Maseru, Lesotho; 2 Ministry of Health, Maseru, Lesotho; 3 EQUIP – Innovation for Health, 1006 Lenchen Avenue North, Centurion, South Africa; 4 Department of Human Nutrition and Dietetics, Sefako Makgatho Health Sciences University, Ga-Rankuwa, Pretoria, South Africa

**Keywords:** HIV care and treatment, Migrants, ART, Lesotho, South Africa, Multi month scripting and dispensing

## Abstract

**Background:**

HIV treatment and care for migrants is affected by their mobility and interaction with HIV treatment programs and health care systems in different countries. To assess healthcare needs, preferences and accessibility barriers of HIV-infected migrant populations in high HIV burden, borderland districts of Lesotho.

**Methods:**

We selected 15 health facilities accessed by high patient volumes in three districts of Maseru, Leribe and Mafeteng. We used a mixed methods approach by administering a survey questionnaire to consenting HIV infected individuals on anti-retroviral therapy (ART) and utilizing a purposive sampling procedure to recruit health care providers for qualitative in-depth interviews across facilities.

**Results:**

Out of 524 HIV-infected migrants enrolled in the study, 315 (60.1%) were from urban and 209 (39.9%) from rural sites. Of these, 344 (65.6%) were women, 375 (71.6%) were aged between 26 and 45 years and 240 (45.8%) were domestic workers. A total of 486 (92.7%) preferred to collect their medications primarily in Lesotho compared to South Africa. From 506 who responded to the question on preferred dispensing intervals, 63.1% (*n* = 319) preferred 5–6 month ARV refills, 30.2% (*n* = 153) chose 3–4 month refills and only 6.7% (*n* = 34) opted for the standard-of-care 1–2 month refills. A total of 126 (24.4%) defaulted on their treatment and the primary reason for defaulting was failure to get to Lesotho to collect medication (59.5%, 75/126). Treatment default rates were higher in urban than rural areas (28.3% versus 18.4%, *p* = 0.011). Service providers indicated a lack of transfer letters as the major drawback in facilitating care and treatment for migrants, followed by discrimination based on nationality or language. Service providers indicated that most patients preferred all treatment services to be rendered in Lesotho, as they perceive the treatment provided in South Africa to be different often less strong or with more serious side effects.

**Conclusion:**

Existing healthcare systems in both South Africa and Lesotho experience challenges in providing proper care and treatment for HIV infected migrants. A need for a differentiated model of ART delivery to HIV infected migrants that allows for multi-month scripting and dispensing is warranted.

**Electronic supplementary material:**

The online version of this article (10.1186/s12889-018-5594-3) contains supplementary material, which is available to authorized users.

## Background

Globally, it is estimated that there were 36.7 [30.8–42.9] million people living with HIV (PLHIV) in 2016, with two thirds from Sub-Saharan Africa [1]. South Africa has the highest number of PLHIV estimated at 7.1 [6.4–7.8] million, although its prevalence of 18.9% [16.6–21.0%] is lower than Swaziland and Lesotho, which bear the highest HIV prevalence estimates in the world at 27.2% [24.9–29.1%] and 25.0% [22.7–26.5%], respectively. Lesotho had 330,000 [300000–360,000] PLHIV in 2016 [2].

The United Nations Joint Programme on HIV and AIDS (UNAIDS) introduced the 90–90-90 targets to reduce the burden of HIV/AIDS globally. This translates to the following targets to be reached by 2020: 90% of all PLHIV will know their HIV status; 90% of all people diagnosed with HIV infection will receive antiretroviral therapy (ART), and 90% of all people on ART will achieve viral suppression [3]. The UNAIDS Report 2017 show that, in Lesotho, 72% of all PLHIV knew their status and 74% of these were on ART [4]. The results of the recent Lesotho Population-based HIV Impact Assessments (PHIA) survey reveal that 68% of PLHIV aged 15–59 years were virally suppressed [5]. While it seems that Lesotho is on track in achieving the 90–90-90 targets, innovative interventions are still needed especially as we are fast approaching the target year of 2020.

To meet the UNAIDS 90–90-90 targets, marginalized subpopulations such as migrants need to be reached. HIV-infected migrants interact with HIV treatment programs and healthcare systems in different countries and this negatively impacts the success of the HIV treatment cascade [6]. HIV treatment requires strict adherence to prevent drug resistance and treatment failure; however, migrants often default in their treatment [7], due to factors such as legal and administrative issues limiting access to treatment for foreign nationals, language and cultural barriers, failure to afford transport costs to collect medications, discrimination from healthcare providers in foreign countries, and the lack of protocols that allow continuity of HIV care across borders of neighbouring countries [8].

The Policy Framework for Population Mobility and Communicable Diseases in the Southern African Development Community (SADC), from which South Africa and Lesotho are member states, acknowledges gaps in plans and strategies for controlling communicable diseases in the region, including HIV/AIDS. The particular gaps and barriers include higher fees for migrants, lack of information on where the services are provided, health care providers not willing to provide long-time treatment such as ART, differing treatment protocols between countries, reluctance of health care providers in dealing with undocumented migrants, and under-resourced health care systems in either source or destination country leading to lack of sufficient drugs [9]. Goal 3 of South Africa’s National Strategic Plan (NSP) for HIV, TB and STIs 2017–2022 is to reach all key and vulnerable populations with customized and targeted interventions, including mobile populations, migrants, and undocumented foreigners. In particular, the proposed interventions include strengthening cross-border collaborations with neighbouring countries and other stakeholders [10]. Furthermore, the South African Constitution provides the right of access to health care to all South African residents including migrants and, in particular, the National Department of Health issued a memo in September 2007 to clarify that refugees and asylum seekers should be given equal access to ART as citizens [11]. However, irrespective of this progressive legislation affording migrants and non-nationals the right to access of health-care, there is lack of effective response to migration and health, especially in Johannesburg, which is home to a diverse of migrants from Southern Africa [12], including Lesotho where most citizens migrate to seek jobs which are not easily available in their country. In this study, we assessed healthcare needs, preferences and accessibility barriers of HIV-infected migrant populations in high HIV burden, borderland districts of Lesotho. Furthermore, we assessed service providers’ knowledge around healthcare services provision to migrant HIV patient populations. The findings of this study are needed to inform the design of targeted interventions that can help Lesotho reach its 90–90-90 targets.

## Methods

### Study design, population and setting

We used a cross-sectional survey design to assess needs, preferences and barriers to HIV care and treatment among HIV-infected migrants from 15 facilities in Lesotho. In this study, we defined a migrant as a Lesotho national who is currently living or has been living in South Africa for at least three consecutive weeks in the past six months.

Lesotho is a small landlocked country surrounded by South Africa. It covers an area of 30,355 km^2^ with an estimated population slightly above 2.2 million in 2017. The gross domestic product per capita for Lesotho was US$2808.20 in 2016 and its economy relies on remittances from migrant labourers who mainly work in neighbouring South Africa. It is divided into 10 districts of which Maseru is the country’s capital city and about 27.3% of the population is urban as of 2015.

**Fig. 1:**
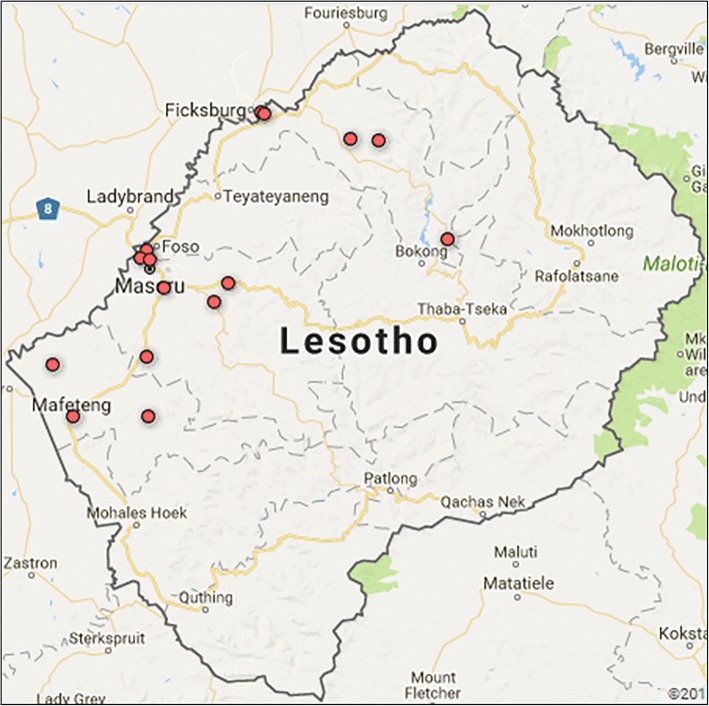
Map of Lesotho showing 15 health facilities included in HIV-infected migrant survey, 2016

We focused on three borderland districts of Lesotho where migration flows are thought to be the highest, targeting high patients’ volume health facilities in Maseru, Leribe and Mafeteng. The geographic distribution of the selected sites in the three districts are indicated by red dots on the map (Fig. 1). The facilities ranged from clinics, health care centres, to hospitals.

### Survey sampling size and sampling allocation

Facilities were selected among high-volume ART sites, using a non-probabilistic quota sampling methodology with proportional allocation across groups, based on district and urban versus rural catchment area.

### Data collection and analysis

HIV-infected individuals who self-identified as migrants were asked to complete the actual survey, which included questions on needs, preferences, and experienced barriers to accessing healthcare services, as well as socio-demographic indicators such as occupation, age and gender. Survey questionnaires were administered within the premises of the 15 selected facilities in the districts of Maseru, Leribe and Mafeteng, by either nurses, pharmacy personnel, or counsellors, depending on the processes in place at each facility. Data collection was undertaken during two consecutive weeks over the Easter holidays, at which time most HIV infected migrants return home to Lesotho to collect their antiretroviral (ARV) medications. As for the qualitative component, purposive sampling was used to recruit health care providers for in-depth interviews across facilities. Two service providers at each facility were identified among those with highest interaction with HIV-positive patients on ART (e.g. clinicians, physicians, nurses, pharmacists, or counsellors). In order to prevent skewed results, only one cadre per type was selected at each facility: this approach ensured sufficient representativeness within the sample of service providers. Service providers as such identified were interviewed in either English or Sesotho by a trained English or Sesotho speaker, according to their preferences.

Data were analysed using STATA version 15 and NVIVO for quantitative and qualitative data, respectively. Descriptive statistics were used to summarize the data, utilizing frequency distribution tables, bar and pie charts to visualize the data. Association between categorical variables was tested using the Chi-square test or Fisher’s exact test, where expected frequency was less than 5. *P*-value of less than 0.05 was considered statistically significant. For the qualitative component, interviews were recorded digitally, and the interviewer captured written notes during the interview. The interviewer used an interview guide and were trained to pursue new themes that came up during the discussion, in order to explore areas of interest to the respondent and the interviewer.

## Results

A total of 2784 HIV-infected patients on ART were surveyed at 15 selected facilities over a period of two weeks (Table 1).

**Table 1:** Sample distribution for HIV-infected migrants enrolled in the study, by site, Lesotho, 2016

District	Location	Health Facility	Patients on ART N	Surveyed N (%)	Migrants N (%)
Leribe	Rural	Matlameng H/C	315	30 (9.5)	7 (23.3)
Leribe	Rural	Pontmain H/C	1337	119 (8.9)	50 (42.0)
Leribe	Rural	Seshote H/C	1136	70 (6.2)	11 (15.7)
Leribe	Urban	Maputsoe Filter Clinic	2074	347 (16.7)	100 (28.8)
Leribe	Urban	Maputsoe SDA H/C	1539	260 (16.9)	15 (5.8)
Mafeteng	Rural	Matelile H/C	667	99 (14.8)	10 (10.1)
Mafeteng	Rural	Ts’akholo H/C	591	235 (39.8)	24 (10.2)
Mafeteng	Urban	Mafeteng Hospital	3277	424 (12.9)	90 (21.2)
Maseru	Rural	Nazareth H/C	1960	441 (22.5)	26 (5.9)
Maseru	Rural	Scott Hospital	1239	136 (11.0)	16 (11.8)
Maseru	Urban	Domiciliary H/C	1550	28 (1.8)	28 (100)^a^
Maseru	Rural	Paki H/C	1185	61 (5.1)	52 (85.2)^a^
Maseru	Urban	RLDF H/C	1247	243 (19.5)	34 (14.0)
Maseru	Rural	St Joseph Hospital	1399	13 (0.9)	13 (100)^a^
Maseru	Urban	Thamae H/C	2178	278 (12.8)	48 (17.3)
TOTAL			**21,694**	**2784 (12.8)**	**524 (18.8)**

^a^H/C – Health Care; At Domiciliary H/C and St Joseph Hospital, surveys were administered only to self-reported migrants; At Paki H/C the same held true, except for the last three days of data collection

Of the total respondents, 524 (18.8%) self-reported as being migrants. Most of the 524 HIV-infected migrants were from Maseru (*n* = 217, 41.4%), followed by Leribe (*n* = 183, 34.9%) and Mafeteng (*n* = 124, 23.7%) districts of Lesotho. The majority were from urban areas (*n* = 315, 60.1%), women (*n* = 344, 65.7%), aged 26–45 years (*n* = 375, 71.6%), and domestic workers (*n* = 240, 45.8%). Rural areas had significantly higher percentage of women (69.4% versus 63.2%, *p* = 0.047) and more domestic workers (58.9% versus 37.1%, *p* < 0.001) than urban areas. There was no significant difference in ages between rural and urban areas (*p* = 0.585) (Table 2).

**Table 2:** Socio-demographics of HIV-infected Lesotho migrants by location

Characteristic	All N (col %)	Urban N (col %)	Rural N (col %)	*P*-value*
Patients	524	315	209	
Sex				0.047
Men	150 (28.6)	101 (32.0)	49 (23.4)	
Women	344 (65.7)	199 (63.2)	145 (69.4)	
Unknown	30 (5.7)	15 (4.8)	15 (7.2)	
Age (years)				0.585
18–25	38 (7.3)	20 (6.3)	18 (8.6)	
26–35	173 (33.0)	105 (33.3)	68 (32.5)	
36–45	202 (38.6)	121 (38.4)	81 (38.8)	
46–55	71 (13.5)	42 (13.3)	29 (13.9)	
55+	17 (3.2)	13 (4.1)	4 (1.9)	
Unknown	23 (4.4)	14 (4.4)	9 (4.3)	
Occupation				< 0.001
Domestic worker	240 (45.8)	117 (37.1)	123 (58.9)	
Construction worker	87 (16.6)	60 (19.0)	27 (12.9)	
Textile worker	52 (9.9)	43 (13.7)	9 (4.3)	
Farmer	24 (4.6)	19 (6.0)	5 (2.4)	
Miner	23 (4.4)	9 (2.9)	14 (6.7)	
Health-care professional	2 (0.4)	2 (0.6)	0 (0.0)	
Student	6 (1.1)	3 (1.0)	3 (1.4)	
Other occupation	49 (9.4)	32 (10.2)	17 (8.1)	
Unknown	41 (7.8)	30 (9.5)	11 (5.3)	

*P-value excludes unknown

Out of the 517 HIV-infected migrants for which defaulting status was known, 126 (24.4%) had defaulted on their treatment. We describe the demographic characteristics by default status (Table 3).

**Table 3:** Socio-demographics of HIV-infected Lesotho migrants by defaulting status

Characteristic	All N	Defaulted N (row %)	Not defaulted N (row%)	*P*-value**
All patients*	517	126	391	
Location				0.011
Rural	206	38 (18.4)	168 (81.6)	
Urban	311	88 (28.3)	223 (71.7)	
District				< 0.001
Leribe	181	34 (18.8)	147 (81.2)	
Mafeteng	123	49 (39.8)	74 (60.2)	
Maseru	213	43 (20.2)	170 (79.8)	
Sex				0.567
Men	148	35 (23.6)	113 (76.4)	
Women	341	89 (26.1)	252 (73.9)	
Unknown	28	2 (7.1)	26 (92.9)	
Age (years)				0.081
18–25	37	3 (8.1)	34 (91.9)	
26–35	172	45 (26.2)	127 (73.8)	
36–45	200	53 (26.5)	147 (73.5)	
46–55	70	19 (27.1)	51 (72.9)	
55+	17	2 (11.8)	15 (88.2)	
Unknown	21	4 (19.0)	17 (81.0)	
Occupation				0.322
Domestic worker	238	67 (28.1)	171 (71.9)	
Construction worker	85	21 (24.7)	64 (75.3)	
Textile worker	52	15 (28.8)	37 (71.2)	
Farmer	23	5 (21.7)	18 (78.3)	
Miner	23	4 (17.4)	19 (82.6)	
Health-care professional	2	2 (100.0)	0 (0.0)	
Student	6	1 (16.7)	5 (83.3)	
Other occupation	49	9 (18.4)	40 (81.6)	
Unknown	39	2 (5.1)	37 (94.9)	

*7 had no default status, ** *P*-value excludes unknown

Default rates were significantly higher in urban areas compared to rural areas (28.3% versus 18.4%, *p* = 0.011). This is also reflected in the differences between districts (*p* < 0.001) due to the different number of urban and rural facilities in the different districts. There was no significant difference in default rates between different genders (*p* = 0.567), ages (*p* = 0.081), and occupation (*p* = 0.322).

**Fig. 2:**
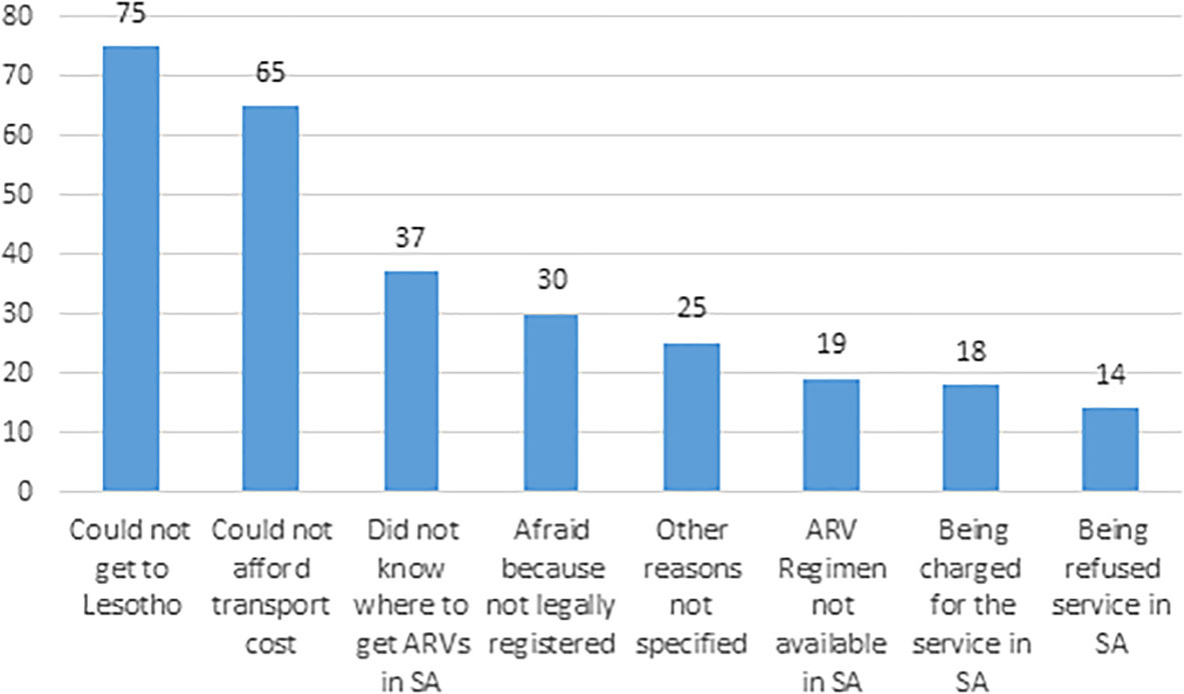
Reasons for defaulting ART amongst HIV infected Lesotho migrants

We also asked the 126 migrants who defaulted regarding their reasons for defaulting on ART while in South Africa: 59.5% (*n* = 75) could not get to Lesotho, 51.6% (*n* = 65) could not afford transport costs, 29.4% (*n* = 37) did not know where to get ARV’s, 23.8% (*n* = 30) were afraid because they were not legally registered, 19.8% (*n* = 25) had other reasons, 15.1% (*n* = 19) said that their ARV regimen was not available at the facility they visited, 14.3% (*n* = 18) were being charged for the ARVs, and 11.1% (*n* = 14) were refused service (Fig. 2).

Out of the 524 HIV infected migrants, the barriers to getting ART while in South Africa included the following: 35.7% (*n* = 187) did not afford transport costs; 23.7% (*n* = 124) did not know where to get ARV’s, 19.3% (*n* = 101) were afraid because of not being legally registered in South Africa; 15.8% (*n* = 83) felt discriminated as a foreigner, 8.6% (*n* = 45) were refused health service; 8.4% (*n* = 44) were afraid that the ARV’s would be confiscated at the border; 6.1% (*n* = 32) had their ARV’s regimen not available at facility; and 5.9% (*n* = 31) had to pay for health services (Table 4).

**Table 4:** Barriers to getting ART in SA among Lesotho migrations

Barrier	N	%
Cannot afford transport costs	187	35.7
Do not know where to get ARVs	124	23.7
No barrier	174	33.2
Afraid if not legally registered in South Africa	101	19.3
Feel discriminated as foreigner	83	15.8
Refused health services	45	8.6
Afraid medications confiscated at the border	44	8.4
ARVs regimen not available at facility	32	6.1
Have to pay for health services	31	5.9

**Fig. 3:**
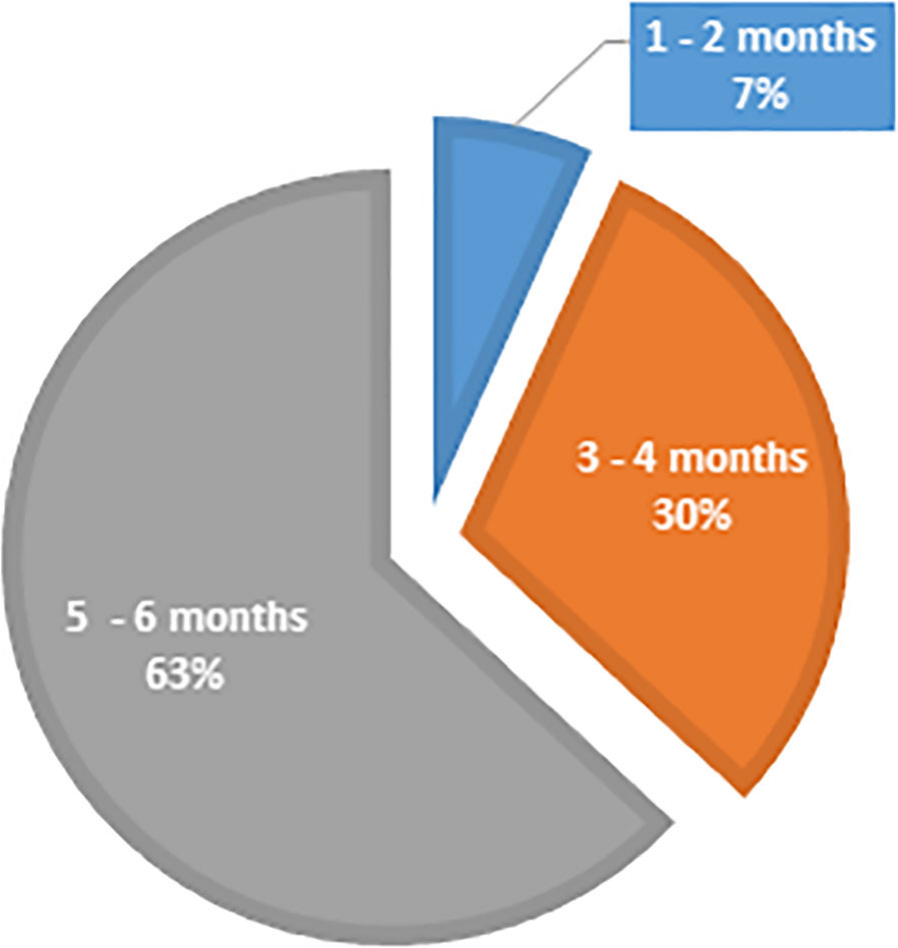
Preferred months of ARV’s refills amongst HIV infected Lesotho migrants

Out of the 524 migrants, 93% (*n* = 486) preferred to collect their medications primarily in Lesotho. From 506 migrants who responded to the question regarding preferred frequency of collecting ARV’s, 6.7% (*n* = 34) opted for the standard-of-care 1–2 month ARV refills whereas 30.2% (*n* = 153) and 63.1% (*n* = 319) indicated a preference for 3–4 month and 5–6 month refills, respectively (Fig. 3).

Quotes under various themes from the in depth interviews with healthcare service providers are summarised below and they confirm and support the quantitative findings.

1. Cultural and nationality-based discrimination
  “They say they even face **discrimination** in some of the health facilities just **because they are foreigners** and some travel very far in order to access services while others do not know where to go at all for example those that work at farms.”
*Pharmacy Technician, St. Joseph hospital, Maseru*
  “Some *[migrant workers]* say that they live very far from the health centres, some believe that the HIV treatment from South Africa is not the same as the one provided in Lesotho (the side effects are extreme), some say that **people from Lesotho are not allowed to access the services**.”
*Nurse, St. Joseph hospital, Maseru*
2. Experienced barriers: Discrimination based on HIV status
  “There are several challenges: most ladies that work as **housekeepers fear possible discriminatory attitudes of their employers** because they know that they will be expelled as soon as they are found to be HIV positive.”
*Pharmacy Technician, St. Joseph hospital, Maseru*
  “They have issues accessing health care services because most of them do not have permanent jobs and they can be fired any time. They have been discriminated against so **disclosing their statuses have become a problem**.”
*Nurse, LDF health centre, Maseru*
3. Experienced barriers: Transport costs
  “They *[migrant workers]* mainly complain about **transport costs** as they come here for refills. They say they **cannot afford to take days off** because it reduces their wages.”
*Professional Counsellor, Maputsoe filter clinic, Leribe*
  “It is mainly **transport costs** and they are always required to present legal documents like passport with work permits.”
*Pharmacy Technician, Domiciliary health centre, Maseru*
4. Experienced barriers: Legal requirements and referring abroad
  “Furthermore, they *[migrant workers]* are required *[to produce]* the **legal documents** and to provide reasons why they are in the foreign countries if they want to access healthcare services abroad and most of the times they do not have such documents.”
*Professional Counsellor, Maputsoe filter clinic, Leribe*
  “Sometimes when they *[migrant workers]* get to the health centres, they are normally required to present some legal documents which allow them to stay in the foreign country and it may happen that some of them might not have it, while others are required to present their ***bukana [health booklets]*** which they may have left behind.”
*Nurse, Matelile health centre, Mafeteng*
  “Some *[migrant workers]* say that they are being **refused to access** their ARV treatment **if they do not present referral letters**.”
*Counsellor, Matelile health centre, Mafeteng*
5. Preferences on ARVs collection site: Perceived treatment differences
  “I normally hear them *[migrant workers]* complaining about the **severe side effects** that are incurred by using ARVs from South Africa. They say that they disfigure their bodies, causing them to have Kyphosis.”
*Counsellor, Matelile health centre, Mafeteng*
  “They *[migrant workers]* prefer Lesotho since there are not as many barriers as in South Africa. Also they believe **the medication from Lesotho is much stronger** than the one supplied in South Africa.”
*Nurse, Paki health centre, Maseru*
6. Current processes for tracking patients who are migrants
  “We use the appointment book to assess whether they are adhering to their appointments because they usually do not give their foreign country of destination contacts. If we need a patient desperately we call their **treatment supporter** and ask them to inform our patients that we would like to see them as soon as possible.”
*Nurse, LDF health centre, Maseru*
  “There are **no specific systems in place** that we use to mark our migrant workers who are on ART.”
*Adherence Counsellor, Tsh’akolo health centre*
7. Health passports and ART road map as new proposed tools for migrants
  “The ART cards are kept by the health facilities and **patients are only provided with health booklets (*bukana*) which contain very brief information** about the patients’ status unlike the ART card.”
*Pharmacist, Mafeteng hospital, Mafeteng*
  “It may be beneficial for them to have their health passport **ready in case of unplanned or sudden migration**.”
*Nurse, Thamae health centre, Maseru*
8. Other proposed interventions for improving migrants’ treatment adherence
  “We should **increase treatment supply** so that they do not come often to the health facilities. Also there should be a well written document focusing on ART patients flow. There should also be a binding legal document between the two countries to curb racism.”
*Adherence Counsellor, LDF health centre, Maseru*
  “I suggest that we provide them with **treatment to last them six months** because they incur so much transport costs having to come for re fills every month.”
*Pharmacy Technician, St. Joseph hospital, Maseru*

## Discussion

The objective of this study was to assess healthcare needs, preferences and accessibility barriers of HIV-infected migrant populations in high HIV burden, borderland districts of Lesotho**.** We surveyed 524 HIV-infected migrants from 15 facilities in the districts of Maseru, Leribe, and Mafeteng. Most of the migrants were from urban areas (60.1%), were women (65.6%), aged 26–45 years (71.6%), and domestic workers (45.8%). Almost a quarter of these migrants (*n* = 126) had defaulted on ART with default rates significantly higher in urban than rural areas. The barriers to getting ART while in South Africa ranged from failing to get to Lesotho, not affording transport costs, not knowing where to get treatment, not being legally registered in South Africa, ARV regimen not being available at facility, and being discriminated against by healthcare providers because they were foreigners. Most of the migrants (93%) preferred to collect their medications primarily in Lesotho and in terms of the frequency of collecting ARV’s, 6.7% opted for the standard-of-care 1–2 month anti-retroviral (ARV) refills whereas 30.2 and 63.1% indicated a preference for 3–4 month and 5–6 month refills, respectively. Service providers indicated a lack of transfer letters, or poor medical history related to ART treatment as the major drawback in facilitating care and treatment for migrants followed by discrimination based on nationality or language. Regarding migrants’ preferred ARVs collection site, service providers indicated that most patients preferred all treatment services to be rendered in Lesotho, as they perceive the treatment provided in South Africa to be different often less strong or with more serious side effects. A need for mHealth systems or telephone tracking to track patients abroad was suggested in combination with multi-month dispensing of ART and harmonizing documentation among neighbouring states, and making referral processes easier.

Several studies on HIV-infected migrants found significantly higher default rates among migrants compared to non-migrants [6], including a community cohort study in Lesotho [7]. Our study found a high default rate of almost 25% among migrants. However we did not collect data to compare with non-migrants. Another study found higher default rates among migrants compared to Spanish born populations (9.2% versus 6.3%) [13]. On the contrary, a study in Johannesburg, South Africa, found default rates to be significantly lower in foreigners compared to local citizens (12% versus 31%) [14]. This implies that our default rate was higher compared to studies from other countries, thereby giving hope that there is potential to reduce the default rate in Lesotho.

The fact that the majority of the migrants were women speaks to the plight of women living with HIV who experience specific barriers to HIV treatment and care because of their gender identity and socially acceptable employment pathways (such as domestic work) within the region’s political economy. The qualitative findings revealed that these women find it difficult to disclose their HIV status for the fear of losing their jobs. As a result, they are unable to request leave to visit health care facilities to access their treatment. There is therefore need to educate the HIV infected migrants regarding their sexual and reproductive health and rights, as well as issues pertaining to gender based violence.

The barriers to receiving ART for migrants have also been well-documented. Barriers that we have identified in this study such as legal and administrative issues, language barriers in communicating in the native language of the host country, and failing to afford transport costs to return to the home country, have also been identified in a review article [6]. However, what is unique about this study is that we have identified that the migrants in Lesotho prefer a multi-month (≥3) supply of medication to cater for them while they are in South Africa. The current status quo is that it is at the discretion of the healthcare professionals to give the migrants ARV’s covering more than the standard-of-care 1–2 months’ supply. The healthcare providers in Lesotho also provide the migrants with transfer letters to allow them to continue their treatment in South Africa; however, most migrants indicated that they do not know where to obtain the ARV’s in South Africa. The fact that 29.7% of the migrants defaulted in ART because they did not know where to get treatment in South Africa speaks to a lack of information that could support migrants in accessing treatment and care when they are away from home. There is therefore need to promote education and distribution of pamplets and notices at country borders to educate migrants regarding where they are able to access treatment. There is also the fear of discrimination due to not being legally documented, coupled with language barriers. Some migrants also claimed that they are refused service in South Africa because of being a foreigner. Some said that they are afraid that their medication would be confiscated at the border between Lesotho and South Africa.

This study had some limitations. As this is a cross-sectional study, we cannot determine cause and effect; and therefore conclusions from this study could be biased. The study is geographically limited to the three districts in Lesotho where most of the migrant population is thought to reside; hence, it may not capture dynamics occurring in other cross-border regions of the country. The assumption was made that rural high-volume sites within a district serve similar populations and that urban high-volume sites within a district serve similar populations as well: should this assumption not hold true, skewed results may occur if the population served by a certain facility presented characteristics that are significantly different from those of the other populations served by facilities in the same group. The study focuses mainly on migrants to South Africa and it is therefore expected not to capture a marginal part of migration flows from Lesotho to other countries. A non-probabilistic sample for selecting high-volume ART facilities raises the possibility that the responses may not be independent and potentially systematically different from a broader group of individuals. However, the large sample size utilized in this study gives it enough statistical power to infer significant associations found in the paper.

## Conclusion

In conclusion, we surveyed HIV-infected migrants in three borderland districts of Lesotho and found a high ART default rate of almost 25%. We also identified barriers to accessing ART, including lack of information on where to get ARVs in South Africa, transport costs of travelling to Lesotho to collect medications, legal and administrative barriers, and discrimination of migrants in foreign countries. Also, most of the migrants preferred collecting their medications in Lesotho and indicated preference of ≥3 months’ supply of ARV refills to cater for when they are in South Africa. Service providers’ perspectives indicate the need to modify and re-structure HIV care among migrants. Specifically, in relation to differentiated model of care that will support multi-month supply of treatment, tracing and mHealth platforms to improve various HIV outcomes including retention, adherence, virologic suppression and ultimately mortality.

We recommend a differentiated model of care specific to HIV infected migrants such as a multi-month scripting and dispensing of treatment. We also recommend the harmonization of treatment protocols for ART between the Lesotho and South African governments and the education and sharing of accessible information across SADC borders on resources, health facilities, and health systems relevant to migrants. We also emphasize that both countries should adhere to the United Nations High Commissioner for Refugees recommendations that ART should not be withheld from displaced persons [14]. We also recommend a qualitative study on the HIV infected migrants to obtain an in-depth understanding of the issues surrounding the barriers to receiving ART.

Since there is such limited data on migrant populations in this SADC region, health systems and HIV treatment and care, we advocate for evidence-based policy change that would meet the healthcare needs of migrant populations in the region. This can include longer-term policy change that align with regional strategies and frameworks, and actions such as training all staff on migration, mobility and health, coordination, and migrant-awareness response to HIV treatment and care.

## Additional file

Additional file 1: Survey data from 524 HIV infected migrants, Lesotho, 2016. (XLSX 44 kb)

## Abbreviations

AIDS: Acquired Immuno-Deficiency Syndrome
ART: Antiretroviral Treatment
ARV: Antiretrovirals
EQUIP: Evaluation and Quality Improvement Program
H/C: Health Centre
HIV: Human Immuno-Deficiency Virus
IOM: International Organization for Migration
NSP: National Strategic Plan
PHIA: Population-based HIV Impact Assessments
PLHIV: People Living with HIV
SADC: Southern African Development Community
SANAC: South African National AIDS Council
UNAIDS: The United Nations Joint Programme on HIV and AIDS
USAID: United States Agency for International Development
